# Effective role of indigenous microorganisms for sustainable environment

**DOI:** 10.1007/s13205-015-0293-6

**Published:** 2015-04-04

**Authors:** Baduru Lakshman Kumar, D. V. R. Sai Gopal

**Affiliations:** Department of Virology, Sri Venkateswara University, Tirupathi, 517502 Andhra Pradesh India

**Keywords:** Indigenous microorganisms, Biodegradation, Bioleaching, Biocomposting, Natural farming, Biofertilizer, Bioremediation

## Abstract

Environmental protection has the foremost importance in the present day life of mankind. Scientists have been researching for technologies naturally available for enhancement of agriculture, management of agricultural waste, etc. Indigenous Microorganisms (IMO’s)-based technology is one such great technology which is applied in the eastern part of world for the extraction of minerals, enhancement of agriculture and waste management. Indigenous microorganisms are a group of innate microbial consortium that inhabits the soil and the surfaces of all living things inside and outside which have the potentiality in biodegradation, bioleaching, biocomposting, nitrogen fixation, improving soil fertility and as well in the production of plant growth hormones. Without these microbes, the life will be wretched and melancholic on this lively planet for the survival of human race. That is why, environmental restoration and safeguarding target via the indigenous microbes in a native manner to turn out the good-for-nothing and useless waste into productive bioresources is the primary concern of this review. Based on the collection sites, the process of collection and isolation methods are different as they may vary from place to place. Ultimately, in this way to a meaningful and significant extent, we can bridge the gap between the horrifying environmental distress and the hostile activities that have been constantly provoked by human kind—by getting these indigenous microorganisms into action.

## Introduction

The uniqueness of microorganisms and their often unpredictable nature and biosynthetic capabilities, given a specific set of environmental and cultural conditions, have made them likely candidates for solving particularly difficult problems in life sciences and other fields as well. The responsible use of indigenous microorganisms to get economic, social and environmental benefits is inherently attractive and determines a spectacular evolution of research from traditional technologies to modern techniques to provide an efficient way to protect environment and new methods of environmental monitoring (Cai et al. 2013). Chemical fertilizers, pesticides, herbicides and other agricultural inputs derived from fossil fuels have increased agricultural production, yet the growing awareness and concern over their adverse effects on soil productivity and environmental quality cannot be ignored. The high cost of these products, the difficulties of meeting demand for them, and their harmful environmental legacy have encouraged scientists to develop alternative strategies to raise productivity, with microbes playing a central role in these efforts (Vaxevanidou et al. 2015). One application is the use of soil microbes as bioinoculants for supplying nutrients and/or stimulating plant growth. Some rhizospheric microbes are known to synthesize plant growth promoters, siderophores and antibiotics, as well as aiding phosphorous uptake. The last 50 years have seen quick steps made in our appreciation of the diversity of environmental microbes and their possible benefits to sustainable agriculture and production. The advent of powerful new methodologies in microbial genetics, molecular biology and biotechnology has only quickened the pace of developments (Patil et al. 2014).

**Fig. 1:**
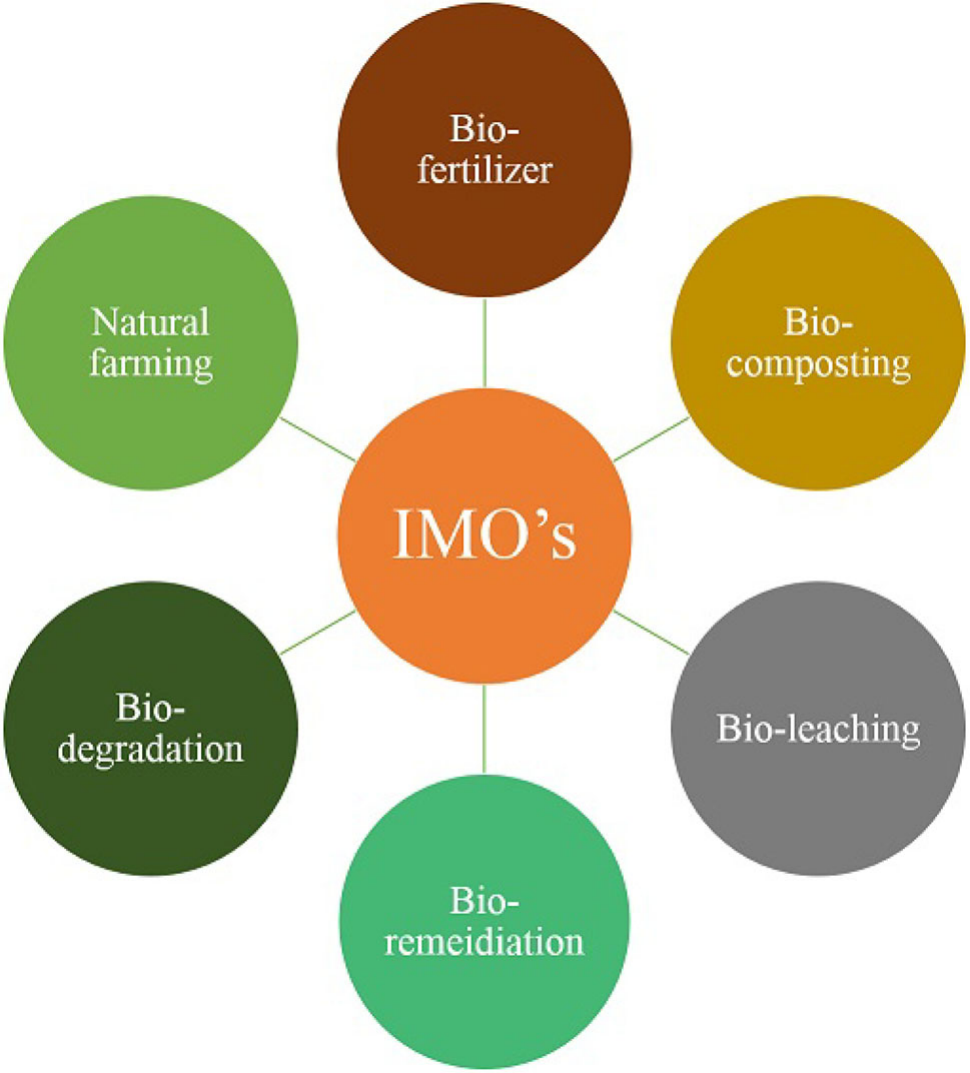
Different application aspects of indigenous microorganisms

The dynamic part played by microbes in sustaining our planet’s ecosystems only adds urgency to this enquiry. Culture-dependent microbes already contribute much to human life, yet the latent potential of vast numbers of uncultured—and thus untouched—microbes, is enormous (Patil et al. 2014). Culture-independent metagenomic approaches employed in a variety of natural habitats have alerted us to the sheer diversity of these microbes and resulted in the characterization of novel genes and gene products. Several new antibiotics and biocatalysts have been discovered among environmental genomes and some products have already been commercialized. Meanwhile, dozens of industrial products currently formulated in large quantities from petrochemicals, such as ethanol, butanol, organic acids and amino acids, are equally obtainable through microbial fermentation (Dong et al. 2011). This review illustrates recent progresses in our understanding of the role of indigenous microbes in sustainable environment (Fig. 1).

## Biodegradation of hydrocarbons

### Role of indigenous microorganisms in remediation of contaminated soils

Degradation of organic compound by indigenous microbes without any artificial enhancement is termed as an “intrinsic bioremediation” and this is one of the best remedial actions for soil contamination. Generally, biodegradation means mineralisation of organic constituents to the soluble inorganic compounds or to transform organic constituents to other soluble organic compounds. In the process of biodegradation of an organic compound, a wide variety of microbial enzymes are involved in transforming both artificial and natural hydrocarbons into intermediate compounds which may be less or equally hazardous than the parental compounds. As biodegradation is a step-wise process, the intermediate compounds are converted into carbon dioxide, water and inorganic compounds which can be readily soluble. Under aerobic conditions, many organic compounds are completely oxidised into soluble inorganic compounds and oxygen acts as a terminal electron acceptor, whereas in anaerobic metabolism the organic compounds are incompletely oxidised into simple organic acids, methane and hydrogen gas as the by-products. Unlike in aerobic metabolism, nitrate, sulphate and bicarbonate act as the terminal acceptors, and the rate of degradation is usually limited by the inherent reaction rate of the active microorganisms.

At a given site, many factors affect the rate of biodegradation process, which are soil moisture, soil pH, availability of oxygen, availability of nutrients, contaminant concentration, and presence of suitable microbes. At higher rates, many hydrocarbons are readily degradable through aerobic metabolism, and only few hydrocarbons are biodegradable through anaerobic metabolism at lower rates. Indigenous microorganisms are inhabited by aquatic as well as oil-bearing deep sub-surface environments (Magot et al. 2000). Oxygen is the key factor that plays a crucial role in the biodegradative process. In relation to this, Aitken et al. (2004, 2008) reported that anaerobic degradation, particularly in methanogenesis, might be the main crude oil biodegradation process in reservoirs. The investigations held by Cai et al. (2013) showed that aerobic indigenous microorganisms also play a role in degrading the petroleum oils. In order to test the efficacy of oxygen/(aerobic microbes) using GC–MS analysis under water flooding wells of Dagang oil fields (oil reservoirs), the reporters initiated the study by partially activating the microbiota via flooding the water channels into oil reservoirs and observed that 99.0 % of *n*-alkanes and 43.03–99.9 % of Polycyclic aromatic hydrocarbons (PAHs) were degraded besides the change in biomarkers to their corresponding ratios, whereas the aerobic culture lasted for 90 days. The metabolite compounds like naphthenic acid, unsaturated fatty acids, aromatic carboxylic acid, alcohols, aldehydes and ketones were separated and identified from aerobic culture. The pathways of alkanes and aromatics were proposed suggests that oxidation of hydrocarbon to organic acid is an important process in the aerobic biodegradation of petroleum.

## Indigenous microorganisms as a biofertilizer

Indigenous microorganisms do not contain a single culture of beneficial microorganisms but a mixture of different beneficial microorganisms; it is a village of good bacteria that are living together in harmony with the rest of nature. The term “indigenous microorganisms” refers to a group of beneficial microbes that are native to the area, thus the name indigenous (locally existing, or not imported); EMs or effective microorganisms on the other hand is a laboratory-cultured mixture of microorganisms. The main difference that divides these two ideas is that IMOs are naturally made, while EMs are man-made, but these two are very much the same with one another in all aspects. IMO-based Technology was actually developed and introduced by Dr. ChouHankyu in 1960s. He employed this technology in natural farms and observed amazing improvements in soil structure and plant health, as the soil upon IMO application regains its loaminess, tilth, structure and even the natural farmer friends, the earthworms, come into droves. Natural farming with IMO Technology is a distinctive approach in organic farming and it has been practised in more than 30 countries in their home gardens and also on a commercial scale. This technology was ritually followed by farmers of Korea, Japan, China, Malaysia, Thailand, Congo, Tanzania, Vietnam, Philippines, and Mongolia. Mr. Chou formulated and fine-tuned these practices and trained over 18,000 people at the Janong Natural Farming institute, south Korea. Later, the other scientist Dr. Hoonpark when in South Korea doing missionary work noticed the commercial piggeries and poultry farms with virtually no stinking smell up on IMO’s usage. This made him to bring IMO Technology to Hawaii. Dr. ChouHankyu has designed and introduced a new eco-friendly farming technology called Indigenous Microorganism technology which is beneficial to the farmers to develop sustainable agriculture and crop production (Cho and Koyama 1997). He developed this technology by the number of experiments conducted in his field studies. Finally, he concludes that natural farming is more advantageous over chemical farming. The techniques and methods employed by Chou are simple, practical, reliable, and economical.

Indigenous microorganisms are a group of innate microbial consortium that inhabits the soil and the surfaces of all living things inside and out which have the potentiality in biodegradation, nitrogen fixation, improving soil fertility, phosphate solubilisers and plant growth promoters (Umi Kalsom and Sariah 2006). Without these microbes, the life will be wretched and melancholic on this lively planet for the survival of human race. That is why, environmental restoration and safeguarding target via the indigenous microbes in a native manner to turn out the good for nothing and useless waste into productive bioresources is the primary concern of this review.

Indigenous microorganisms play an important role by protecting the normal host from invasion by microorganisms with a greater potential for causing disease. They compete with the pathogens for essential nutrients and for receptors on host cells by producing bacteriocins and other inhibitory substances, making the environment inimical to colonization by pathogens. They are the important component of world biodiversity (Sadi et al. 2006). These microorganisms increase the availability of nutrients to host plants (Vessey 2003) and increase the water-holding capacity, making the plants to have sufficient (or) enough water all the time. It improves the aeration to the plant roots such that exchange of gases takes place effectively and prevents soil erosion. Based on the collection sites, the process of collection and isolation methods are different as they may vary from place to place. Many environmental factors affect the rate of biodegradation potential and this involves both physical and chemical factors such as temperature, pH, organic matter, oxygen availability, availability of nutrients and so on. Indigenous microorganisms in each stage of composting were isolated and screened for the abilities to solubilise phosphate and produce indole-3-acetic acid (IAA). The potential microorganisms were selected for development of biofertilizer. The modified “Natural Farming” composting method is a simple, cheap, and fast method used to produce EFB compost that contains beneficial microorganisms with the potential to be developed into biofertilizer. Phua et al. (2011) particularly studied on the isolated indigenous microorganisms which may enhance plant growth through N2 fixation using the 15 N isotopic tracer technique, solubilise insoluble inorganic phosphate compounds or hydrolyse organic phosphate to inorganic P or stimulate plant growth through hormonal actions such as IAA production. Combination of microbial strains could be a good multifunctional biofertilizer for sustainable agriculture. The result derived from the IAA test showed that two isolates were IAA producers and six isolates produced clear zones on phosphate agar plates indicating phosphate-solubilising activity. Agro-waste management and enhancement of biodiversity are the approaches towards sustainability (Shukor 2009; Ong 2009).

Han et al. (2006) also showed that the combined treatment of *Bacillus megaterium* var. *phosphaticum* and *B. mucilaginosus* increased the availability of phosphorus and potassium in soil, and thus increasing the uptake and plant growth of pepper and cucumber. Sarma et al. (2009) reported that a combination of bioinocculents, namely, two *Fluorescent* *pseudomonas* strains, increased *Vigna mungo* yield by 300 % in comparison to the control crop. These results indicated that a combination of beneficial microorganisms might increase the nutritional assimilation of plant and total nitrogen in soil.

IMOs create the optimum and favourable environment to improve and maintain soil flora and soil fauna as well as the other microorganisms which in turn support the quality life of higher plants and animals including the human. Soil particles are lumped in aggregates and fostered to provide air and water retention, which in turn creates a good habitat for other symbiotic microbes. The IMOs are eco-friendly, environmentally safe and healthy with potential to create hunger-free environment. Better quality crops and livestock are assured due to the absence of synthetic chemical fertilizers and pesticides as inputs.

## Bioleaching of heavy metals by indigenous microorganisms

For decades, concerned companies and local authorities have demonstrated interest in managing water from metallurgical and mining areas. After processing of mineral ores in metallurgical industries, the residual metals in effluent water would cause a substantial loss in revenue for metallurgical companies. Secondly, in view of environmental safety, these metals are potential pollutants of the water system. According to the South African National Standard (2005), an excess of calcium and iron in water could cause aesthetic or operational problems, while excess of magnesium in water could cause aesthetic and health problems. The process of removal of metals from the solutions through the physico-chemical techniques is called chemical leaching and if this process occurs through microorganisms, it is called bioleaching which can be done by indigenous, exogenous and genetically manipulated microorganisms. Compared to costly physico-chemical techniques, the biological techniques are found to be cheap and eco-friendly (Kefala et al. 1999; Cohen 2006; Alluri et al. 2007). Generally, metal removal efficiency greatly depends on the affinity between the metal and the microbial cell wall, and this can be achieved using indigenous microorganism isolated around mine areas.

Attempts are made at laboratory scale to remediate the high concentration of calcium, iron and magnesium in surface waters from metallurgical areas, using indigenous strains of* Shewanella* sp,* Bacillus subtilis* sp and *Brevundimonas* sp, which revealed variable abilities of these microorganisms in the removal process. Living and non-living biomass of all these strains will be tested for their effectiveness to clean out Ca, Mg and Fe predominant in surface water around mining areas in Nigel.

The inherent abilities of microorganisms are suitable for the removal of metals from solutions (Beveridge and Murray 1976; Langley and Beveridge 1999; Nies 1999). These abilities have been identified as passive or active for accumulation and biosorption, respectively, (Brandl and Faramarzi 2006). Indigenous strains are more suitable to overcome the challenges such as high concentration of metals, acidic conditions as they had adapted to conditions in situ. Bacillus strains have been widely used in the removal of metals (Pb, Cd, Cu, Ni, Co, Mn, Cr, Zn) from wastewaters (Philip and Venkobachar 2001; Srinath et al. 2003; Kim et al. 2007).

### Metal removal

Metals are removed from the solutions through passive biosorption and active bioaccumulation. In biosorption, an anionic group (amino acids, hydroxyl, phosphate, etc.) on the bacterial cell membrane binds to the positively charged metal in solution where no energy is required during this process. Whereas in bioaccumulation, the metal is sequestered through the bacterial membrane into the cytoplasm of the cell; during this process, microorganisms use energy (ATP hydrolysis) to catalyse the reaction.

### Bioleaching of heavy metals from sewage sludge using indigenous iron-oxidising microorganisms

Due to rapid population increase in the world, generation of potentially contaminated heavy metals is expected to be more in the sewage sludge than in the metropolitan cities. Prior to land application, traditional chemical methods such as EDTA extraction and acid treatment have been suggested for solubilisation of heavy metals from the sludge, but EDTA showed low removal efficiencies for Fe, Ni and Cr(Jenkins et al. 1981). The high cost and large consumption of chemical agents have made chemical methods unattractive. To overcome these problems, heavy metals in sewage sludge are removed through bioleaching process, the recently emerged strategy which is economical, easy operation, and non- hazardous to products (Babel and del Mundo Dacera 2006; Pathak et al. 2009). The experimental batch system studies by Xiang et al. (2000) showed that the isolated indigenous iron-oxidising bacteria have more ability in reducing the heavy metals from anaerobically digested sewage sludge from the Yuen Long wastewater treatment plant, HongKong. In this study, to maintain low pH and to accelerate the solubilisation of Cr, Cu, Zn, Ni and Pb, FeSo_4_ is added along with the isolated indigenous microorganisms. After 16 days of bioleaching, more than 80 % of Cu, Zn, Ni and Cd are removed as compared with that of the control. Similarly, Wen et al. (2013) studies on leaching kinetics show that solubilisation of heavy metals or removal efficiency is more by the indigenous iron-oxidising bacteria than without using indigenous microorganisms in the control ones which were collected from the Fuzhou Jingshan sewage water plant. In this study, after isolation of bacteria from the sludge, they were enriched by Wen et al. (2013) medium for three generations before the use of inoculum for bioleaching experiment (Ren et al. 2009). Solid concentration, pH, inoculum concentration, and FeSo_4_ effectively influence the bioleaching of metals. Effectiveness depends upon the metal species because of their different bindings in sludge; removal of Zn from the sludge was dominated by chemical leaching, while the removal of Cu, Pb and Cr was dominated by bioleaching (Wen et al. 2013).

The two species *Acidithiobacillus ferrooxidans* and *Acidithiobacillus thiooxidans* have a great significance in bioleaching process (Tyagi et al. 1993; Chan et al. 2003). Here iron-based bioleaching is considered to be superior to sulphur-based bioleaching due to sludge acidification and heavy metal solubilisation in sulphur-based bioleaching (Wong and Gu 2008). To leach successfully, ammonium ferrous sulphate and ferrous sulphate are used to enrich the indigenous iron-oxidising microorganisms in sludge with a neutral pH (Pathak et al. 2009). Metal removal from dewatered metal plating sludge using *A. ferrooxidans* indicates that pH, oxidation–reduction potential (ORP), sulphate production, pulp density and agitation time were all important parameters in bioleaching (Bayat and Sari 2010).

## Bioremediation of oil spills

Oil spills are treated as a widespread problem that poses a great threat to any ecosystem. Bioremediation has emerged as the best strategy for combating oil spills and can be enhanced by the following two complementary approaches: bioaugmentation and biostimulation. In bio augmentation, the addition of oil-degrading bacteria boosts biodegradation rates, whereas in biostimulation, the growth of indigenous hydrocarbon degraders is stimulated by the addition of nutrients (mainly N and P) or other growth-limiting nutrients (Nikolopoulou and Kalogerakis 2010). Crude oil is composed of a wide range of different compounds, which makes it difficult for the indigenous population to cope with this broad variety of substrates, and hence oil-degrading microorganisms could be added to supplement the indigenous population (Leahy and Colwell 1990). There is increasing evidence that the best approach for overcoming these barriers is the use of microorganisms from the polluted area. A new concept in bioaugmentation, known as "autochthonous bioaugmentation" (ABA), has been proposed by Ueno et al. (Ueno et al. 2007) and is defined as a bioaugmentation technology that exclusively uses microorganisms indigenous to the sites (soil, sand, and water) slated for decontamination. The success of oil spill bioremediation depends on the establishment and maintenance of physical, chemical and biological conditions that favour enhanced oil biodegradation rates in the marine environment. Through Biostimulation, the growth of indigenous oil degraders is stimulated by the addition of nutrients (nitrogen and phosphorous) or other growth-limiting co-substrates and/or by alterations in environmental conditions (e.g., surf-washing, oxygen addition by plant growth, etc.). The document issued by the Natural Biodegradation as a Remedial Action Option Interim Guidance (1994) illustrated the capabilities of an acclimated indigenous microbial consortium sampled from a pristine environment in the presence or absence of other rate-limiting factors (i.e., nutrients and biosurfactants) (biostimulation) as a potential strategy for the successful remediation of polluted marine environments (http://www.dnr.wi.gov/files/pdf/pubs/rr/rr515.pdf). Effectiveness of autochthonous bioaugmentation together with biostimulation versus biostimulation- only strategies for the successful remediation of polluted marine environments.

Many reports show that ozonation is effective in removing contaminants such as polyhydrocarbons in diesel-contaminated soils. (Hus and Masten 1997; Lim et al. 2002; Stehr et al. 2001; Sung and Huang 2002). As ozone has strong oxidising activity, it reacts with organic compounds to form oxidised products which are more or water soluble and bioavailable than parental compounds leading to a better biodegradation (Legube et al. 1981; Gilbert 1983). There are some reports given previously about the external introduction of microorganisms into ozonated soil (Nam and Kukor 2000; Stehr et al. 2001). But this external inoculation does not show promising result and caused a rapid decline of cell number and/or activity of inoculated cells (Van Veen et al. 1997). Therefore, employing already-acclimatised indigenous microorganisms could be an alternative to achieve successful remediation. Based on these ideas, (Ahn et al. 2005) developed the Pre-Ozonation and subsequent bioremediation technology for better degradation of polyhydrocarbons in the contaminated soils. Prolonged ozonation of diesel-contaminated soils for 0–900 min not only decreases the pH but also the viable indigenous bacterial concentrations. So, to increase the microbial number, the ozonated soils are incubated for 9 weeks and monitored by conventional culture-based methods (Plate count and Phenanthrene spray plate assay) and non-culture-based molecular methods (direct soil DNA extraction and catabolic gene probing). All taken together, this study is the first to monitor the potential of indigenous microorganisms to degrade pH in Ozonated soil.

### Bioremediation of 2,4-D in soils using nanoparticles Fe_3_O_4_ and indigenous microbes

Fang et al. (2012) studied the degradation kinetics of 2,4-D in soils by Fe_3_O_4_ nanoparticles and indigenous microbes. In this study, the degradation efficiency of 2,4-D was investigated in three different treatments: indigenous microbes, Fe_3_O_4_ nanoparticles, or combined use of Fe_3_O_4_ nanoparticles and indigenous microbes. The results indicated that the combined use of Fe_3_O_4_ nanoparticles and indigenous microbes led to greater mineralisation of 2,4-D to its degradation products, 2,4-DCP,chlorophenol (2-CP, 4-CP) and phenol, than the treatments with Fe_3_O_4_ nanoparticles or indigenous microbes alone, and is more advantageous in remediation of soil contaminated with herbicides.

### Arsenic mitigation

Arsenic (As) is a notorious toxic metalloid which is of geogenic and anthropogenic origin. It is prevalent in many regions of the world (Mandal and Suzuki 2002; Tripathi et al. 2007). Generally, inorganic arsenic species are believed to be more toxic than organic forms to living organisms, including humans and other animals (Goessler 2002; Meharg and Hartley‐Whitaker 2002). Microbes developed various intrinsic As tolerance mechanism to sustain in the adverse environmental conditions. Metal-resistant bacteria often have genes located on plasmids. Genetic system named ars operon is the main functional unit for As resistance (Mukhopadhyay and Rosen 2002). The toxicity of different arsenic species was often reported to vary in an order of arsenite > arsenate > mono-methylarsonate (MMA) > dimethylarsinate (DMA) (Penrose and Woolson 1974; Sturgeon et al. 1989).

Bioremediation of As by microorganisms has been widely hailed because of its cost-effective process and eco-friendly in nature (Valls and Lorenzo 2002). Conversion of metalloids to their volatile derivatives by organisms is a well-known phenomenon in nature (Challenger 1945). During arsenic volatilization, some species of fungi and bacteria methylate inorganic As species to relatively less-toxic volatile methylarsenicals (Rodriguez et al. 1999; Cernansky et al. 2009).

The investigations by Majumder et al. (2013) shows that the indigenous bacterial isolates AMT-08 and AGH-09 showed higher As volatilizing capacity under aerobic conditions; ADP-18 volatilized maximum As under anaerobic conditions, while AMT-04 performed well in both conditions. The bacterial strains isolated from arsenic-contaminated soils have shown much higher resistance to As volatalization than those isolated from soil, gold mines, and geothermal effluents in the related researches throughout world (Saltikov and Olson 2002; Simeonova et al. 2004). AMT-08 has been found most successful in managing the removal of the metalloid from soil throughout the entire incubation period; it removes 10 % (in 30 days) and 16 % (in 60 days) As when supplemented with farm yard manure. The strain AMT-08 has an increased As volatilizing ability with exogenous nutrients (farm yard manure) and successful exploitation of these hyper-tolerant isolates may deliver an eco-friendly tool for As mitigation within manageable expenditure as compared to the genetically engineered ones.

The bacterial strains under the present investigation were isolated from anaerobic soil environment (submerged paddy soil) which is predominated by AsIII over AsV and hence showed much higher tolerance to AsIII as compared to findings from related research established as a model microorganism for bioremediation of arsenic and one of the most arsenic-resistant microorganisms (400 mM for AsV and 60 mM for AsIII) described to date (Mateos et al. 2010).

## Role of indigenous microorganisms in natural farming

Natural farming is the propagation of mycorrhizae, by adding specific inputs during the nutritive cycle of the plant. Mycorrhizae are “fungus roots” which act as an interface between plants and soil. They grow into the roots, increasing the root system many thousands of times over. They act symbiotically and convert the complex substrates to simpler ones. Miles of fungal filaments can be present in an ounce of healthy soil. Mycorrhizal inoculation of soil increases the accumulation of carbon by depositing glomalin, which in turn increases soil structure by binding organic matter to mineral particles in the soil. Natural farming with IMO is a distinctive approach to organic farming practised successfully in more than 30 countries, in home gardens and on a commercial scale. Amazing improvements have been seen in soil structure and plant health, as upon application of indigenous microorganisms in natural farming the soil regains its loaminess, tilth and structure, and the earthworms come in droves. Mr Cho formulated and fine-tuned these practices for 40 years and has trained over 18,000 people at the Janong Natural Farming Institute (janonglove.com), and the dedicated work of Dr. Hoon Park brought Natural Farming to Hawaii. Dr. Park was in South Korea doing missionary work and noticed commercial piggeries with virtually no smell that were using Natural Farming methods. Mr. Cho has spread Natural Farming worldwide and planted the trees in Gobi Desert, Mongolia but had failed three times earlier, under the harsh wind and with only few inches of rainfall a year. With Natural Farming methods, the trees had a 97 percent survival rate and are now 20 feet tall. Corn and barnyard grasses have been planted for livestock feed, and wells have been dug. Watermelon farming now provides a stable income to farmers there also. As he learned more about these practices, he realized that they could help eliminate hunger and poverty in extremely poor parts of the world.

### Cultivation of indigenous microorganisms

- IMO-1—Cultured in a simple wooden box of rice;
- IMO-2—Mixed with brown sugar and stored in a crock;
- IMO-3—Further propagated on rice bran or wheat mill run;
- IMO-4—Mixed with clay soil/ant hill soil;

The result is then mixed with compost, added to potting soil, or spread on beds before planting. The entire process takes three to 4 weeks. Other inputs and sprays are made from fermented plant juices, made from the tips of growing plants mixed with brown sugar. There are also recipes for water-soluble calcium made from eggshells, fish amino acid made from fish waste, lactic acid bacteria and insect attractants made from rice wine. There is also water-soluble calcium phosphate made from animal bones which are used according to the nutritive/growth cycle of the plants. The fish amino acids are simply fresh fish waste, de-boned and packed into a container with brown sugar and fermented for a few months. The pigs’ excrement is so odourless, clean and dry, that you literally do not even have to clean it out. The benefits of using the Natural Farming methods include the following:

- Lower costs to the farmer (by as much as 60 percent)
- More desirable crops
- Stronger, healthier and more nutritious plants
- Higher yield
- Better quality
- Farmer friendly
- Zero waste emission.

The inputs are made from natural materials, which are not only safe for the environment, but actually invigorate and rehabilitate the ecology.

## Biocomposting by indigenous microorganisms

Agro-industrial wastes have become a major problem in terms of disposal because most farmers dispose them through burning. This inexpensive method of crop residue disposal is practised in many parts of the world to clear the excess residue from land for faster crop rotation, control undesirable weeds, pests and diseases and to return some nutrient to the soil. Open burning emits a large amount of harmful air pollutants (particles and inorganic and organic gases), which have severe impact on human health, polycyclic aromatic hydrocarbons (PAHs) (Korenaga et al. 2001) and the danger of soil erosion due to repeated burning (Kahlon and Dass 1987). In addition, disposal in water bodies (for example, river or lake) may contribute to a decrease in water quality. Because of these concerns, there is a need to find efficient alternatives for agro-industrial waste management. Many alternatives for the disposal of these residues have been proposed, composting being one of the most attractive on account of its low environmental impact and cost (Bustamante et al. 2008; Canet et al. 2008; Lu et al. 2009) as well as its capacity for generating a valuable product used for increasing soil fertility (Weber et al. 2007) or as a growing medium in agriculture and horticulture (Perez‐Murcia et al. 2005). To manage Paddy husk and Corn Stalk residues through composting, a study was conducted by Hanim et al. (2012) to determine the physical and chemical properties of different composts and humic acid extracted from the final product. For this study, they took paddy husk, corn stalk and kitchen waste with different concentrations as the substrate (organic source) and to this they added the IMO compost in six different treatments i.e. T_1_ to T_6_.and the results showed that IMO compost from Corn stalk had better quality (chemical characteristics) compared to that of paddy husk.

## Summary and conclusion

Currently, environmental sustainability is a contemporary issue that receives plenty of attention from the research scientists. This is a result of the amount of research going into assessing the impact that human activity can have on the environment. Although the long-term implications of this serious issue are not yet fully understood, it is generally agreed that the risk is high enough to merit an immediate response. As stated earlier, indigenous microorganisms-based technology is one such important technology and these organisms inhabit the soil with the abilities of biodegradation, bioleaching, biocomposting, nitrogen fixation, improving soil fertility and as well in the production of plant growth hormones. In addition, these are large group of naturally occurring and often unknown or ill-defined microorganisms that interact favourably in soils and with plants to render beneficial effects which are sometimes difficult to predict. Indigenous microorganisms usually denote specific mixed cultures of known, beneficial microorganisms that are being used effectively as microbial inoculants that could exist naturally in soil or added as microbial inoculants to soil where they can improve soil quality, enhance crop production and create a more sustainable agriculture and environment. IMOs coexist and are physiologically compatible and mutually complementary, and if the initial inoculum density is sufficiently high, there is a high probability that these microorganisms will become established in the soil and will be effective as an associative group, whereby such positive interactions would continue. If so, then it is also highly probable that they will exercise considerable control over the indigenous soil microflora in due course. Still lot of constructive research is required to make use of IMOs in sustainable environment.

## References

[CR1] Ahn Y, Jung H, Tatavarty R, Choi H, Yang JW, Kim IS (2005). Monitoring of petroleum hydrocarbon degradative potential of indigenous microorganisms in ozonated soil. Biodegradation.

[CR2] Aitken RJ (2005). Founders’ lecture. Human spermatozoa: fruits of creation, seeds of doubt. Repro Fert Develop.

[CR3] Aitken CM, Jones DM, Larter SR (2004). Anaerobic hydrocarbon biodegradation in deep subsurface oil reservoirs. Nature.

[CR4] Aitken CM, Jones DM, Head IM, Gray ND, Adams JJ, Rowan AK, Aitken CM, Bennett B, Huang H, Brown A, Bowler BFJ, Oldenburg T, Erdmann M, Larter SR (2008). Crude-oil biodegradation via methanogenesis in subsurface petroleum reservoirs. Nature.

[CR5] Alluri HK, Ronda SR, Settalluri VS, Bondili JS, Suryanarayana V, Venkateshwar P (2007). Biosorption: An eco-friendly alternative for heavy metal removal. Afr J Biotechnol.

[CR6] Babel S, del Mundo Dacera D (2006). Heavy metal removal from contaminated sludge for land application: a review. Waste Manage.

[CR7] Bayat B, Sari B (2010). Comparative evaluation of microbial and chemical leaching processes for heavy metal removal from dewatered metal plating sludge. J Hazard Mater.

[CR8] Beveridge TJ, Murray RG (1976). Uptake and retention of metals by cell walls of *Bacillus subtilis*. J Bacteriol.

[CR9] Brandl H, Faramarzi MA (2006). Microbe-metal-interactions for the biotechnological treatment of metal-containing solid waste. Chin Particuol.

[CR10] Bustamante M, Paredes C, Marhuenda-Egea F, Pérez-Espinosa A, Bernal M, Moral R (2008). Co-composting of distillery wastes with animal manures: carbon and nitrogen transformations in the evaluation of compost stability. Chemosphere.

[CR11] Cai M, Yao J, Yang H, Wang R, Masakorala K (2013). Aerobic biodegradation process of petroleum and pathway of main compounds in water flooding well of Dagang oil field. Bioresour Technol.

[CR12] Canet R, Pomares F, Cabot B, Chaves C, Ferrer E, Ribó M, Albiach MR (2008). Composting olive mill pomace and other residues from rural southeastern Spain. Waste Manage.

[CR13] Cernansky S, Kolencik M, Sevc J, Urik M, Hiller E (2009). Fungal volatilization of trivalent and pentavalent arsenic under laboratory conditions. Bioresour Technol.

[CR14] Challenger F (1945). Biological methylation. Chem Rev.

[CR15] Chan L, Gu X, Wong J (2003). Comparison of bioleaching of heavy metals from sewage sludge using iron-and sulfur-oxidizing bacteria. Adv Environ Res.

[CR16] Cho HK, Koyama A (1997) Korean natural farming: indigenous microorganisms and vital power of crop/livestock. Korean Natural Farming

[CR17] Cohen RRH (2006). Use of microbes for cost reduction of metal removal from metals and mining industry waste streams. J Clean Prod.

[CR18] Dong Y, Butler EC, Philp RP, Krumholz LR (2011). Impacts of microbial community composition on isotope fractionation during reductive dechlorination of tetrachloroethylene. Biodegradation.

[CR19] Fang G, Si Y, Tian C, Zhang G, Zhou D (2012). Degradation of 2, 4-D in soils by Fe3O4 nanoparticles combined with stimulating indigenous microbes. Environ Sci Pollut Res.

[CR20] Gilbert E (1983). Investigations on the changes of biological degradability of single substances induced by ozonation. Ozone Sci Engrg.

[CR21] Goessler W, Kuehenelt D (2002) Analytical methods for the determination of arsenic and arsenic compounds in the environment. In: William T, Frankenberger Jr William (eds) CRC Press, pp 27–50

[CR22] Han HS, Supanjani E, Lee KD (2006). Effect of co-inoculation with phosphate and potassium solubilizing bacteria on mineral uptake and growth of pepper and cucumber. Plant Soil Environ.

[CR23] Hanim AN, Muhamad AN, Ahmed OH, Susilawati K, Khairulmazmi A (2012). Physico-chemical properties of indigenous microorganism-composts and humic acid prepared from selected agro-industrial residues. Afr J Biotechnol.

[CR24] Hus I, Masten SJ (1997). The kinetics of the reaction of ozone with phenanthrene in unsaturated soils. Environ Eng Sci.

[CR25] Jenkins D, Wolever T, Taylor RH, Barker H, Fielden H, Baldwin JM, Bowling AC, Newman HC, Jenkins AL, Goff DV (1981). Glycemic index of foods: a physiological basis for carbohydrate exchange. Am J Clin Nutr.

[CR26] Jones D, Head I, Gray N, Adams J, Rowan A, Aitken C, Bennett B, Huang H, Brown A, Bowler B (2007). Crude-oil biodegradation via methanogenesis in subsurface petroleum reservoirs. Nature.

[CR27] Kahlon SS, Dass SK (1987). Biological conversion of paddy straw into feed. Biol Wastes.

[CR28] Kefala MI, Zouboulis AI, Matis KA (1999). Biosorption of cadmium ions by actinomycetes and separation by flotation. Environ Pollut.

[CR29] Kim SU, Cheong YH, Seo DC, Hur JS, Heo JS, Cho JS (2007). Characterisation of heavy metal tolerance and biosorption capacity of bacterium strain CPB4 (*Bacillus* spp.). Water SciTechnol J Int Asso Water Pollut Res.

[CR30] Korenaga T, Liu X, Huang Z (2001). The influence of moisture content on polycyclic aromatic hydrocarbons emission during rice straw burning. Chemos Glob Change Sci.

[CR31] Langley S, Beveridge TJ (1999). Effect of O-side-chain-lipopolysaccharide chemistry on metal binding. Appl Environ Microbiol.

[CR32] Leahy JG, Colwell RR (1990). Microbial degradation of hydrocarbons in the environment. Microbiol Rev.

[CR33] Legube B, Langlais B, Sohm B, Dore M (1981). Identification of ozonation products of aromatic hydrocarbon micropollutants: effect on chlorination and biological filtration. Ozone Sci Eng: J Int Ozone Asso.

[CR34] Lim HN, Hawng TM, Kang JW (2002). Characterization of Ozone decomposition in a soil slurry: kinetics and mechanisms. Water Res.

[CR35] Lu Y, Wu X, Guo J (2009). Characteristics of municipal solid waste and sewage sludge co-composting. Waste Manage.

[CR36] Magot M, Ollivier B, Patel BK (2000). Microbiology of petroleum reservoirs. Antonie Van Leeuwenhoek.

[CR37] Majumder A, Bhattacharyya K, Kole S, Ghosh S (2013). Efficacy of indigenous soil microbes in arsenic mitigation from contaminated alluvial soil of India. Environ Sci Pollut Res.

[CR38] Mandal BK, Suzuki KT (2002). Arsenic round the world: a review. Talanta.

[CR39] Mateos LM, Ordóñez E, Letek M, Gil JA (2010). Corynebacterium glutamicum as a model bacterium for the bioremediation of arsenic. Int Microbiol.

[CR40] Meharg AA, Hartley-Whitaker J (2002). Arsenic uptake and metabolism in arsenic resistant and nonresistant plant species. New Phytol.

[CR41] Mukhopadhyay R, Rosen BP (2002). Arsenate reductases in prokaryotes and eukaryotes. Environ Health Persp.

[CR42] Nam K, Kukor JJ (2000). Combined ozonation and biodegradation for remediation of mixtures of polycyclic aromatic hydrocarbons in soil. Biodegradation.

[CR43] Nies DH (1999). Microbial heavy-metal resistance. Appl Microbiol Biotechnol.

[CR44] Nikolopoulou M, Kalogerakis N, Timmis KN (2010). Biostimulation strategies for enhanced bioremediation of marine oil spills including chronic pollution. Handbook of hydrocarbon and lipid microbiology.

[CR45] Ong HK (2009) Agro-waste management: Approaches towards sustainability. In: 2nd National Conference on Agro-environment 2009 Johor Bahru, Johor, pp 73–81

[CR46] Pathak A, Dastidar M, Sreekrishnan T (2009). Bioleaching of heavy metals from sewage sludge by indigenous iron-oxidizing microorganisms using ammonium ferrous sulfate and ferrous sulfate as energy sources: a comparative study. J Hazard Mater.

[CR47] Patil SS, Adetutu EM, Rochow J, Mitchell JG, Ball AS (2014). Sustainable remediation: electrochemically assisted microbial dechlorination of tetrachloroethene-contaminated groundwater. Microb Biotechnol.

[CR48] Penrose W, Woolson E (1974). Arsenic in the marine and aquatic environments: analysis, occurrence, and significance. Crit Rev Environ Sci Technol.

[CR49] Perez-Murcia MD, Moreno-Caselles J, Moral R, Perez-Espinosa A, Paredes C, Rufete B (2005). Use of composted sewage sludge as horticultural growth media: effects on germination and trace element extraction. Comm Soil Sci Plant Anal.

[CR50] Philip L, Venkobachar C (2001). An insight into the mechanism of biosorption of copper by Bacillus polymyxa. Int J Environ Pollut.

[CR51] Phua CKH, Wahid ANA, Rahim A (2011). Development of multifunctional bio fertilizer formulation from indigenous microorganisms and evaluation of Their N2-fixing capabilities on chinese cabbage using 15 N tracer technique. Pertanika J Trop Agric sci.

[CR52] Ren WX, Li PJ, Zheng L, Fan SX, Verhozina VA (2009). Effects of dissolved low molecular weight organic acids on oxidation of ferrous iron by *Acidithiobacillus ferrooxidans*. J Hazard Mater.

[CR53] Rodriguez RR, Basta NT, Casteel SW, Pace LW (1999). An in vitro gastrointestinal method to estimate bioavailable arsenic in contaminated soils and solid media. Environ Sci Technol.

[CR54] Sadi T, Jeffey LSH, Rahim N, Rashdi AA, Nejis NA, Hassan R (2006) Bio prospecting and management of microorganisms. National conference on agro biodiversity conservation and sustainable utilization, pp 129–130

[CR55] Saltikov C, Olson B (2002). Homology of *Escherichia coli* R773 arsA, arsB, and arsC genes in arsenic-resistant bacteria isolated from raw sewage and arsenic-enriched creek waters. Appl Environ Microbiol.

[CR56] Sarma MVRK, Saharan KPA, Bisaria VS, Sahai VS (2009). Application of fluorescent pseudomonads inoculant formulations on *Vigna mungo* through field trial. World Acad Sci Eng Technol.

[CR57] Shukor AAR (2009) Quality agro-environment: the key to productive and sustainable agriculture. In: 2nd National Conference on Agro-environment 2009, Johor Bahru, Johor. pp 3–8

[CR58] Simeonova D, Lievremont D, Lagarde F, Muller D, Groudeva V, Lett MC (2004). Microplate screening assay for detection of AsIIIoxidizing and AsV-reducing bacteria. FEMS Microbiol Lett.

[CR59] Srinath T, Garg SK, Ramteke PW (2003). Biosorption and elution of chromium from immobilized Bacillus coagulans biomass. Ind J Exp Biol.

[CR60] Stehr J, Müller T, Svensson K, Kamnerdpetch C, Scheper T (2001). Basic examinations on chemical pre-oxidation by ozone for enhancing bioremediation of phenanthrene contaminated soils. Appl Microbiol Biotechnol.

[CR61] Sturgeon RE, Siu KM, Willie SN, Berman SS (1989). Quantification of arsenic species in a river water reference material for trace metals by graphite furnace atomic absorption spectrometric techniques. Analyst.

[CR62] Sung M, Huang CP (2002). In situ removal of 2-chlorophenol from unsaturated soils by Ozonation. Environ Sci Technol.

[CR63] Tripathi RD, Srivastava S, Mishra S, Singh N, Tuli R, Gupta DK, Maathuis FJ (2007). Arsenic hazards: strategies for tolerance and remediation by plants. Tren Biotechnol.

[CR64] Tyagi RD, Blais JF, Auclair JC, Meunier N (1993). Bacterial leaching of toxic metals from municipal sludge: influence of sludge characteristics. Water Environ Res.

[CR65] Ueno A, Ito Y, Yumoto I, Okuyama H (2007). Isolation and characterization of bacteria from soil contaminated with diesel oil and the possible use of these in autochthonous bioaugmentation. World J Microbiol Biotechnol.

[CR66] Umi KMS, Sariah M (2006) Utilization of microbes for sustainable agriculture in Malaysia: current status. Bio prospecting and management of microorganisms. National Conference on Agro biodiversity conservation and sustainable utilization, pp 27–29

[CR67] Valls M, Lorenzo V (2002). Exploiting the genetic and biochemical capacities of bacteria for the remediation of heavy metal pollution. FEMS Microbiol Rev.

[CR68] Van Veen JA, van Overbeek LS, van Elsas JD (1997). Fate and activity of microorganisms introduced into soil. Microbiol Mol Biol Rev.

[CR69] Vaxevanidou K, Christou C, Kremmydas GF, Georgakopoulos DG, Papassiopi N (2015). Role of indigenous arsenate and iron (III) Respiring microorganisms in controlling the mobilization of arsenic in a Contaminated soil Sample. Bull Environ Cont Toxicol.

[CR70] Vessey JK (2003). Plant growth promoting rhizobacteria as biofertilizers. Plant Soil.

[CR71] Weber J, Karczewska A, Drozd J, Licznar M, Licznar S, Jamroz E, Kocowicz A (2007). Agricultural and ecological aspects of a sandy soil as affected by the application of municipal solid waste composts. Soil Biol Biochem.

[CR72] Wen YM, Cheng Y, Tang C, Chen ZL (2013). Bioleaching of heavy metals from sewage sludge using indigenous iron-oxidizing microorganisms. J Soil Sed.

[CR73] Wong JWC, Gu XY (2008). Optimization of Fe^2+^/solids content ratio for a novel sludge heavy metal bioleaching process. Water Sci Technol.

[CR74] Xiang L, Chan L, Wong J (2000). Removal of heavy metals from anaerobically digested sewage sludge by isolated indigenous iron-oxidizing bacteria. Chemosphere.

